# Ultrasound-guided peripheral nerve blocks for osteoarthritis pain management: a review of the genicular, suprascapular, PENG and deep peroneal nerve blocks

**DOI:** 10.3389/fmed.2026.1942434

**Published:** 2026-09-22

**Authors:** Susana Almeida, Anita Cunha, Bruno Daniel Carneiro, José Tavares-Costa, Filipa Teixeira

**Affiliations:** 1 Rheumatology Department, Unidade Local de Saúde do Alto Minho, Ponte de Lima, Portugal; 2 Unit of Experimental Biology, Department of Biomedicine, Faculty of Medicine, University of Porto, Porto, Portugal

**Keywords:** deep peroneal nerve block, genicular nerve block, osteoarthritis, PENG block, peripheral nerve block, suprascapular nerve block, ultrasound-guided

## Abstract

**Background:**

Osteoarthritis (OA) is the most prevalent form of arthritis and a leading cause of chronic pain worldwide. A significant proportion of patients experience refractory pain despite optimized conservative treatment, underscoring the need for alternative analgesic strategies. Ultrasound-guided peripheral nerve blocks targeting joint-specific nociceptive pathways have been proposed as a minimally invasive option, yet the evidence across different joints has not been comprehensively appraised.

**Objective:**

This narrative review summarizes the available evidence on the therapeutic use of ultrasound-guided peripheral nerve blocks across four targets in OA: the genicular nerves (knee), the suprascapular nerve (glenohumeral joint), the pericapsular nerve group — PENG (hip), and the deep peroneal nerve (ankle/midfoot), examining anatomical rationale, ultrasound-guided technique, clinical efficacy, safety, and current gaps.

**Results:**

The genicular nerve block has the most robust evidence base, with multiple placebo-controlled randomized trials (RCTs) and meta-analyses demonstrating significant pain relief sustained for up to 24 weeks, though overall certainty remains low to very low by GRADE criteria. The suprascapular nerve block is supported by one placebo-controlled RCT and a meta-analysis of broader chronic shoulder pain populations showing sustained benefit up to 12 weeks, with ultrasound-guided approaches yielding consistently larger effect sizes. The PENG block has one RCT comparing it with intra-articular injection: the PENG block provided superior pain relief at day 1, whereas the intra-articular group showed better pain and functional outcomes at weeks 4 and 8; both groups achieved significant pain reduction through 8 weeks. Evidence for the deep peroneal nerve block is limited to retrospective case series confirming high rates of immediate pain relief but lacking controlled data. Across all blocks, no serious adverse events or motor deficits were reported, and the role of corticosteroid adjuvants remains uncertain. Substantial heterogeneity in techniques, injectate protocols, and outcome measures limits cross-study comparison.

**Conclusion:**

In selected patients with refractory OA pain, ultrasound-guided peripheral nerve blocks may offer analgesic benefit with a favorable safety profile, best considered as part of a multimodal approach. However, well-designed sham-controlled RCTs with standardized protocols and extended follow-up are needed to define their efficacy, optimal technique, and positioning within the OA treatment algorithm.

## 1 Introduction

Osteoarthritis (OA) is the most prevalent form of arthritis and a common cause of chronic pain and long-term disability worldwide. In 2020, an estimated 595 million individuals were affected, corresponding to 7.6% of the global population, with projections indicating a continued rise driven by population aging and increasing obesity rates. Although OA most commonly affects the knee and hip, any joint can be involved (1). The disease is currently recognized as a whole-joint process involving cartilage, subchondral bone, synovium, ligaments, and periarticular tissues, resulting from a complex interplay of mechanical, inflammatory, and metabolic factors (2). Pain — its cardinal symptom — is multifactorial in nature: initially nociceptive and mechanical, it may evolve into chronic pain through peripheral and central sensitization mechanisms, with a neuropathic component present in 20%–40% of knee OA and 9%–29% of hip OA patients (3). This neurophysiological complexity underpins the growing interest in interventional approaches targeting joint innervation.

Conventional management follows a stepwise strategy combining non-pharmacological measures — exercise, patient education, and weight management — with pharmacological therapy, including non-steroidal anti-inflammatory drugs (NSAIDs) and intra-articular corticosteroids, as recommended by major international guidelines (4). For glenohumeral and ankle OA, however, no high-quality joint-specific guidelines exist, and recommendations are largely extrapolated from knee and hip evidence (5, 6). Moreover, the mainstays of pharmacological treatment carry relevant limitations. NSAID use is constrained by gastrointestinal, cardiovascular and renal adverse effects, particularly in the elderly and multimorbid patients typical of OA populations, and expert recommendations advise against frequent repeated intra-articular corticosteroid injections, given uncertainty about their long-term structural effects on cartilage (4, 7).

Guideline coverage of nerve-targeted therapies in OA is limited to the knee and to ablative techniques: the American College of Rheumatology (ACR) conditionally recommends radiofrequency ablation (8), and the American Academy of Orthopaedic Surgeons (AAOS) states that denervation therapy may reduce pain and improve function, although this recommendation was downgraded for inconsistent evidence (9). By contrast, neither the Osteoarthritis Research Society International (OARSI) nor the most recent European Alliance of Associations for Rheumatology (EULAR) recommendations for hip and knee OA cover nerve-targeted interventions (10, 11). Local anesthetic peripheral nerve blocks are not specifically addressed by any of these societies.

More broadly, no available therapy has demonstrated disease-modifying effects (7), and existing interventions offer limited long-term efficacy (12). A significant proportion of patients experience refractory pain despite optimized conservative treatment, and many are not candidates for or do not wish to undergo joint replacement surgery (7), underscoring the need for alternative analgesic strategies.

In this context, ultrasound-guided peripheral nerve blocks have been proposed as a minimally invasive strategy targeting joint-specific nociceptive pathways. By depositing local anesthetic, with or without corticosteroid, adjacent to articular nerve branches, these procedures interrupt nociceptive signaling at the joint level while minimizing systemic exposure.

This review aims to summarize the available evidence on the therapeutic use of ultrasound-guided peripheral nerve blocks across four targets with emerging evidence in OA—the genicular nerves in knee OA, the suprascapular nerve (SSN) in glenohumeral OA, the pericapsular nerve group (PENG) in hip OA, and the deep peroneal nerve in ankle/midfoot OA—examining anatomical rationale, technique, efficacy, safety, and current gaps in the literature.

## 2 Ultrasound-guided nerve blocks for osteoarthritis

### 2.1 Genicular nerve block

#### 2.1.1 Anatomy and rationale

The knee joint receives sensory innervation from a complex and overlapping network of articular branches arising from the femoral (FN), sciatic, and obturator nerves, with considerable anatomical variability and no consensus on the precise number, origin, and trajectory of individual branches (13, 14); the anterior knee alone is supplied by up to 10 nerves derived from these three trunks, with 12–13 contributing to the joint overall (15). The anterior capsule exhibits the highest nociceptor density and is considered the principal anatomical target for knee pain interventions. Moreover, it appears to be the only knee structure supplied by all three parent nerves—the FN (via the saphenous and the nerves to the vastus medialis and intermedius), the sciatic, and the obturator nerves (13).

Among these articular branches, interventional practice has centered on three genicular branches that follow a consistent periosteal course at the junction of the femoral shaft with the respective epicondyles—superior medial genicular nerve (SMGN) and superior lateral genicular nerve (SLGN)—and of the tibial shaft with the medial epicondyle—inferior medial genicular nerve (IMGN). Each genicular nerve is accompanied by a corresponding genicular artery, a relationship that provides the anatomical basis for ultrasound-guided periarterial targeting (14, 16).

The classical three-target paradigm, first described by Choi et al. in 2011 for radiofrequency neurotomy, targets SMGN, SLGN and IMGN at their periosteal course (16). This approach has since been adopted for diagnostic and therapeutic blockade with local anesthetic. The inferior lateral genicular nerve (ILGN) has traditionally been excluded from the target set owing to the proximity of its course to the common fibular nerve trunk near the fibular head (14). However, these three targets represent only a fraction of the nerves supplying the knee, and their position varies between individuals relative to classic bony and radiographic landmarks, such that a three-target approach cannot address the full innervation of the joint; capturing all articular nerves may nonetheless be unnecessary, provided those mediating an individual patient's pain are targeted (15).

#### 2.1.2 Ultrasound-guided procedure

The block is performed with the patient in the supine position and the knee slightly flexed over a pillow (17). The most commonly described ultrasound-guided technique uses a periarterial approach, in which the genicular arteries — easily visualized with Doppler, unlike the small nerves themselves—serve as sonographic surrogates for the adjacent genicular nerves. Alternative techniques include bony landmarks at the diaphysis-epicondyle junction, where the nerve runs adjacent to bone, as well as soft tissue structures, dynamic maneuvers, and fixed anatomical reference lines (18). Using the periarterial approach, a transducer is placed sequentially over the medial distal femur, the lateral distal femur, and the proximal medial tibia to identify the superior medial, superior lateral, and inferior medial genicular arteries, respectively, which serve as surrogate landmarks for the SMGN, SLGN, and IMGN (18). Needle placement is confirmed by real-time visualization of the needle tip reaching the periosteum, with some protocols using hydrolocation or Doppler confirmation of the accompanying genicular artery (18).

Cadaveric injection studies have identified precise bony targets for the SMGN (1 cm anterior to the adductor tubercle) and IMGN (midpoint between the tibial medial epicondyle and the tibial insertion of the medial collateral ligament), with 100% accuracy confirmed on dissection (19).

The ILGN has traditionally been avoided due to the risk of damaging the common fibular nerve, which can result in iatrogenic foot drop (18). Fonkoué et al. proposed a distal pre-capsular target at a safe distance from the common fibular nerve trunk—at the intersection of a line through Gerdy's tubercle and one through the tibial tuberosity, derived from cadaveric dissection but not yet validated under ultrasound guidance (14).

Most studies use 2–3 mL of local anesthetic per target nerve, alone or combined with a corticosteroid, although considerable heterogeneity exists across studies regarding agent, concentration, and adjuvant use (18, 20, 21).

#### 2.1.3 Clinical evidence

A meta-analysis of six randomized controlled trials (RCTs) (420 participants) in primary and secondary knee OA reported that genicular nerve block (GNB) significantly reduced pain (standardized mean difference, SMD = 1.00) and improved function (SMD = 1.07) compared with various control interventions, with peak pain relief at 2 weeks and maximal functional improvement at 12 weeks (22). A separate meta-analysis of 25 RCTs (2,049 participants) evaluating minimally invasive genicular nerve interventions (including GNB, radiofrequency ablation, and cryoneurolysis) applied GRADE methodology and rated the evidence for GNB as very low to low across all comparisons, citing risk of bias, inconsistency and imprecision (23).

Among the RCTs included in these analyses, only Shanahan et al. used a sham control: 64 patients were randomized to ultrasound-guided GNB (bupivacaine plus betamethasone) or saline placebo, with significant improvements in visual analogue scale (VAS) pain at weeks 2, 4, and 8, with diminution of effect by week 12 (VAS 4.6 vs. 5.1; *p* = 0.055); Western Ontario and McMaster Universities Osteoarthritis Index (WOMAC) total scores remained significantly improved through week 12, with a progressively narrowing between-group difference (17). Two further placebo-controlled RCTs, published after these meta-analyses, have since strengthened the evidence base. Binici et al. randomized 81 patients to GNB with prilocaine, prilocaine plus triamcinolone, or placebo, reporting significantly greater improvements in VAS in both active groups compared with placebo at 1 week and 1 month (*p* < 0.01); WOMAC total scores also improved significantly in both active groups by 1 month, though only the corticosteroid group reached significance at 1 week (21). De la Fuente-Escudero et al. randomized 102 patients to bupivacaine plus triamcinolone, bupivacaine alone, or saline placebo, showing that both active groups achieved significantly greater numeric rating scale (NRS) pain reduction than placebo (interaction *p* = 0.001) sustained at 24 weeks. WOMAC pain improved in both active groups at early timepoints but lost significance by 24 weeks; WOMAC physical function improvement was sustained only in the bupivacaine-alone group (interaction *p* = 0.007), with the corticosteroid group returning to baseline at 24 weeks (20).

Regarding corticosteroid adjuvants, Kim et al. found that adding triamcinolone to lidocaine produced lower VAS scores at 2 and 4 weeks (*p* < 0.001), but the clinically meaningful benefit lasted only 2 weeks with scores in both groups returning to baseline by 8 weeks (24). Two subsequent placebo-controlled RCTs found no significant differences between local anesthetic alone and anesthetic plus corticosteroid in direct between-group comparisons (20, 21).

No serious adverse events or motor deficits were reported across available RCTs, consistent with the motor-sparing profile of selective periosteal targeting. Minor self-limiting events included transient injection-site pain and isolated hypoesthesia (20–23).

### 2.2 Suprascapular nerve block

#### 2.2.1 Anatomy and rationale

The suprascapular nerve (SSN) is a mixed sensorimotor nerve arising from the superior trunk of the brachial plexus (C5–C6), providing motor innervation to the supraspinatus and infraspinatus muscles and sensory innervation to the posterior glenohumeral capsule, the acromioclavicular and coracoclavicular ligaments, and the subacromial bursa (25, 26). The SSN supplies most of the sensory innervation to the shoulder joint, with the remaining contribution arising from articular branches of the axillary, lateral pectoral, and subscapular nerves (27).

From its origin, the SSN courses laterally beneath the trapezius muscle and enters the supraspinous fossa through the suprascapular notch, a bony canal roofed by the superior transverse scapular ligament, above which the suprascapular artery passes (26). The SSN gives off three sequential sensory branches: the medial subacromial branch (MSAb), arising proximal to the suprascapular notch; the lateral subacromial branch (LSAb), arising at the level of the suprascapular notch; and the posterior glenohumeral branch (PGHb), arising distal to the spinoglenoid notch (25). Distal to the spinoglenoid notch, the nerve terminates in motor branches to the infraspinatus (26).

Considerable anatomical variability has been described along the course of the SSN. The superior transverse scapular ligament varies in thickness and may be bifid or calcified in 15% and 10% of cases, respectively, while the suprascapular artery shows numerous variants and courses within the notch itself in 2.5% of cases (26). The morphology of the suprascapular notch is likewise variable (28). The origin of the sensory branches relative to the notch is also inconsistent, with the MSAb arising proximal to the notch in the majority of individuals (25).

Although articular branches of the axillary, lateral pectoral, and subscapular nerves also contribute to shoulder innervation and have been proposed as potential targets for radiofrequency ablation, no RCTs have evaluated their blockade for chronic shoulder pain, and no widely accepted protocols exist for ablation targeting (29). To the best of our knowledge, the SSN block is therefore the only peripheral nerve block technique with placebo-controlled trial evidence in degenerative shoulder disease.

Given this innervation pattern, the location of pain may inform patient selection: the SSN block is anatomically best suited for posterior and superior shoulder pain, whereas predominantly anterior pain — mediated by articular branches of the axillary, lateral pectoral and subscapular nerves — may be less likely to respond to isolated SSN blockade (27, 29). Nevertheless, pain in glenohumeral OA is often diffuse rather than well localized, and the SSN remains the rational primary target given its predominant contribution to joint innervation.

#### 2.2.2 Ultrasound-guided procedure

Two main ultrasound-guided approaches to the SSN block have been described. The posterior (distal) approach targets the nerve at the floor of the supraspinous fossa, between the suprascapular notch and the spinoglenoid notch, where the supraspinatus fascia confines the injectate close to the nerve. With the patient seated or in prone position, a linear transducer is placed in a coronal plane over the suprascapular fossa with a slight anterior tilt, until the suprascapular notch is identified (30). Notch morphology influences the visualization of the structures in this region and may require adjustment of transducer positioning. At this level, the pulsating suprascapular artery serves as the sonographic key for the adjacent nerve (28). A needle is advanced until the tip reaches the floor of the supraspinous fossa, close to the suprascapular notch, and correct needle tip placement is confirmed by visualization of injectate spread deep to the supraspinatus fascia (30). Injection proximal to the suprascapular notch is recommended to maximize blockade of all three sensory branches of the SSN, as the MSAb arises proximal to the notch in most cases and would otherwise be spared (25).

An alternative supraclavicular (proximal) approach, first described by Siegenthaler et al. in 2012, targets the SSN at its origin from the superior trunk of the brachial plexus, beneath the omohyoid muscle in the posterior cervical triangle. This site offers superior ultrasound visualization and a more superficial nerve course compared with the distal fossa target (31). However, its proximity to the brachial plexus carries a clinically relevant risk of phrenic nerve blockade — 40% incidence of hemidiaphragmatic paralysis vs. 2% with the posterior approach (32). For this reason, the posterior approach is generally preferred.

In a systematic review of 53 studies on SSN block for chronic shoulder pain, bupivacaine was the most commonly used long-acting local anesthetic, at 0.25%–0.5% concentration and volumes ranging from 1 to 10 mL, and corticosteroid was added in over half of the studies, most commonly methylprednisolone 40 mg (33).

#### 2.2.3 Clinical evidence

Direct evidence for the SSN block in glenohumeral OA remains limited. The only placebo-controlled RCT including patients with degenerative shoulder disease was conducted by Shanahan et al., who randomized 83 patients (108 shoulders) with chronic shoulder pain — 52% from degenerative disease and 48% from rheumatoid arthritis — to a single-shot SSN block (10 mL bupivacaine 0.5% with methylprednisolone 40 mg) or subcutaneous saline placebo. The SSN block produced significant improvements in Shoulder Pain and Disability Index (SPADI) pain and disability scores at 1, 4, and 12 weeks compared with placebo, with 67% of treated shoulders achieving clinically meaningful improvement (≥10 points SPADI reduction) at week 1 versus 23% with placebo (*p* < 0.01), sustained at 55% versus 18% at week 12 (*p* < 0.01) (34).

Indirect evidence from broader chronic shoulder pain populations supports these findings. A meta-analysis of 11 RCTs (591 patients) with heterogeneous shoulder pathologies and comparators found that the SSN block provided superior pain relief compared with placebo (SMD = 0.70 at 12 weeks) and physical therapy (SMD = 0.75 at 12 weeks), with efficacy comparable to intra-articular corticosteroid injection. In subgroup analysis by guidance technique, only ultrasound-guided approaches showed consistent improvements across all timepoints (SMD = 0.95 at 12 weeks) compared with landmark- and fluoroscopy-based techniques (35).

The role of corticosteroid adjuvants remains unclear. A recent systematic review of five RCTs with at least 3-month follow-up found that studies using local anesthetic combined with corticosteroid reported significant pain reduction at 3 months, whereas those using local anesthetic alone did not achieve the same efficacy — although this reflects an indirect comparison across heterogeneous studies rather than a head-to-head trial (36). A multisociety practice guideline confirmed that no RCT has directly compared the two regimens for this block (37).

The safety profile of the SSN block appears favorable. In a systematic review of 111 studies, among the 3,122 participants who received SSN block injections, the incidence of physical harm was low: local pain and bruising (1.35%), transient motor weakness (0.83%), and paresthesia (0.54%). Pneumothorax (0.16%) occurred only in association with concurrent multiple-site landmark-guided injections rather than the SSN block itself. Peripheral nerve injury (0.10%) was reported in a single landmark-guided study, with recovery at eight weeks. No nerve injury was reported with ultrasound-guided approaches and no cases of local anesthetic systemic toxicity were reported. However, the quality of harms assessment and reporting across studies was generally poor (38).

### 2.3 PENG block

#### 2.3.1 Anatomy and rationale

The sensory supply of the hip capsule is complex and variable. Three nerves consistently contribute — articular branches of the femoral and obturator nerves to the anterior capsule, and the nerve to the quadratus femoris to the posterior capsule — with inconsistent accessory contributions from the accessory obturator, superior gluteal, sciatic, and inferior gluteal nerves. The anterior capsule shows the highest nociceptor density, rendering it the principal pain generator in hip OA, whereas the posterior capsule is comparatively sparsely innervated by nociceptors (39).

Building on this neuroanatomical framework, Girón-Arango et al. first described the PENG (PEricapsular Nerve Group) block in 2018, targeting the musculofascial plane between the psoas tendon and the superior pubic ramus at the level of two consistent bony landmarks — the anterior inferior iliac spine (AIIS) and the iliopubic eminence (IPE) — where the articular branches of the FN, and the accessory obturator nerve when present, course toward the anterior capsule (40). By depositing local anesthetic in this pericapsular interfascial plane rather than around the main nerve trunks, the PENG block aims to provide selective sensory blockade with potential motor preservation, an advantage over FN block and fascia iliaca block, both of which carry a risk of quadriceps weakness (40). Because it targets the anterior pericapsular plane, the PENG block would not be expected to cover pain of posterior capsular origin.

#### 2.3.2 Ultrasound-guided procedure

The block is performed with the patient in the supine position. An ultrasound transducer is placed transversely over the AIIS and rotated approximately 45° to align with the superior pubic ramus. In this view, the key sonoanatomic landmarks are identified from lateral to medial: the AIIS, the IPE, and the superior pubic ramus, with the hyperechoic psoas tendon overlying the bone. The femoral artery should be identified medially to ensure it is avoided during needle advancement (40). A needle is advanced in-plane from lateral to medial allowing continuous needle visualization, until the tip reaches the musculofascial plane between the psoas tendon and the pubic ramus (40, 41). Correct placement is confirmed by hydrolocation and visualization of injectate spreading deep to the psoas tendon, lifting it away from the bone (40).

In the available OA-specific studies, the injectate consisted of a long-acting local anesthetic (ropivacaine or bupivacaine) at variable volumes, with dexamethasone added in one trial (40–42).

#### 2.3.3 Clinical evidence

Evidence for the PENG block in hip OA remains limited. The only RCT comparing the PENG block with an active comparator in hip OA was conducted by Kose et al., who randomized 60 patients to either a single-shot PENG block or ultrasound-guided intra-articular injection, both using the same mixture (bupivacaine 0.25% with dexamethasone 8 mg) (43). The PENG block provided superior early pain relief at day 1 (NRS 2.57 vs. 4.80; *p* < 0.001), whereas the intra-articular group demonstrated better pain scores at week 4 (NRS 2.60 vs. 3.50; *p* < 0.001) and week 8 (NRS 3.10 vs. 4.80; *p* < 0.001), as well as better Harris Hip Score (HHS) at weeks 4 and 8 (*p* = 0.021 and *p* = 0.007, respectively), and better WOMAC scores at week 8 (*p* = 0.036). Both groups achieved significant pain reduction up to 8 weeks, with no between-group difference in analgesic consumption or patient satisfaction (43).

De Andrade Filho et al. conducted a prospective up-and-down study in 26 patients with hip OA, estimating the minimum effective volume for 50% of patients (MEV₅₀) of the PENG block at 1.53 mL of local anesthetic when combined with a standardized obturator nerve block. Quadriceps strength was preserved — and even improved — in all patients with successful blocks, reinforcing the motor-sparing profile at low volumes (42).

However, cadaveric and clinical studies using higher volumes have reported FN spread and quadriceps weakness in up to 75% of cases, suggesting that the motor-sparing advantage is volume-dependent (44, 45).

The safety profile of the PENG block appears favorable: no major complications were reported across available OA-specific studies (42, 43).

The current evidence base is limited to one RCT with a maximum follow-up of 8 weeks (43) and one dose-finding study (42), both with small sample sizes and no sham-controlled design.

### 2.4 Deep peroneal nerve block

#### 2.4.1 Anatomy and rationale

The deep peroneal nerve (DPN), also known as the deep fibular nerve, is a terminal branch of the common peroneal nerve that courses in the anterior compartment of the leg superficial to the anterior tibial artery, between the tibialis anterior and extensor hallucis longus muscles (46). It provides motor innervation to the anterior compartment muscles responsible for foot and toe dorsiflexion, a consideration relevant to the safety profile of DPN blockade. At ankle level, the DPN contributes to the sensory innervation of the anterior capsule of the talocrural joint, alongside branches of the saphenous and superficial peroneal nerves (47). It then crosses deep to the extensor hallucis longus tendon to enter the interval between the extensor hallucis longus and extensor digitorum longus tendons, coursing superficial to the anterior joint capsule, before bifurcating into a medial sensory branch — which follows the dorsalis pedis artery and crosses the talonavicular joint capsule toward the midfoot — and a lateral motor branch that penetrates the extensor digitorum brevis (46). Because the motor branches to the anterior compartment muscles arise proximally in the leg, blockade at the distal leg lies distal to these branches, preserving dorsiflexor function. Distally, its terminal sensory branches consistently innervate the dorsal capsules of the 2nd and 3rd tarsometatarsal (TMT) joints, with variable innervation of the naviculocuneiform, 4th TMT, 1st TMT, and 5th TMT joints (48).

The DPN thus represents the primary nociceptive pathway of the dorsal midfoot and a contributor to the anterior talocrural capsule, making it a rational therapeutic target in OA of these regions, consistent with Hilton’s law — the principle that nerves supplying muscles acting on a joint also provide sensory innervation to that joint.

This anatomical distribution provides the rationale for selectively targeting the DPN rather than performing a complete regional ankle block, and its superficial course in the anterior distal leg makes it a readily accessible target for ultrasound-guided blockade.

To the best of our knowledge, the DPN is the only nerve target at the ankle and midfoot for which clinical data on peripheral nerve blockade in OA are available.

#### 2.4.2 Ultrasound-guided procedure

The patient is positioned supine with the knee slightly flexed and the sole of the foot resting on the table. A transducer is placed transversely over the anterior distal leg, approximately 3–4 cm proximal to the ankle joint (49). At this level, the anterior tibial artery and its paired veins serve as the primary sonographic landmarks for identifying the DPN, which appears as a hypoechoic dot or cluster of fascicular bundles surrounded by echogenic epineurium (49). The position of the DPN relative to the anterior tibial artery is variable: in a retrospective series of 25 ultrasound-guided DPN blocks, the nerve was located lateral to the artery in 48% of cases, anterior in 20%, medial in 12%, lateral to the paired veins in 16%, and 1 cm lateral to the artery in 4% (49). Given this variability, real-time ultrasound guidance is essential to accurately identify the nerve and adapt the needle approach accordingly.

This proximal approach — performed at the anterior leg rather than at the ankle level — is preferred in the context of OA because the block targets the DPN proximal to its bifurcation, thereby covering articular branches to both the ankle and midfoot simultaneously (49). Using an in-plane technique, a needle is advanced to the outer edge of the nerve epineurium, and local anesthetic is injected perineurally to achieve circumferential spread (49).

Available clinical series of isolated DPN block for midfoot OA have used a combination of lidocaine and bupivacaine. Johnston et al. performed ultrasound-guided DPN blocks in the anterior leg using lidocaine 1% for perineural hydrodissection to separate the epineurium from surrounding soft tissues, followed by 2.5–3 mL of bupivacaine 0.5%–0.75% deposited perineurally (49); Shi et al. used 2 mL of lidocaine 1% and 2 mL of bupivacaine 0.5% using a conventional landmark technique (48).

#### 2.4.3 Clinical evidence

Evidence for DPN blockade in OA is limited to small retrospective observational studies, with no RCTs currently available. The most relevant direct evidence derives from two retrospective series focusing on dorsal midfoot OA. Shi et al. performed diagnostic DPN blocks using a landmark-based technique in 37 patients with radiographically confirmed dorsal midfoot OA refractory to conservative treatment, reporting a mean immediate pain improvement of 92.1%, confirming that dorsal midfoot nociceptive pain is at least partially transmitted by the DPN (48). Johnston et al. described the ultrasound-guided technique in the anterior leg in 25 feet (22 with midfoot OA), reporting pain relief in 88% (22/25), assessed either immediately post-injection or at a subsequent clinic visit (49). In both studies, blocks were performed with local anesthetic alone for diagnostic purposes, and no structured follow-up of block duration was reported.

No data exist on the role of corticosteroid addition to local anesthetic in DPN blocks.

Taken together, the available evidence supports DPN blockade as a diagnostic tool to confirm DPN-mediated nociceptive pain in dorsal midfoot OA with potential—yet unproven—therapeutic value as a standalone intervention.

No adverse effects were reported by Shi et al. (48). Johnston et al. reported no immediate complications but one patient developed DPN irritation at the injection site at 2 months, without evidence of intraneural injection, and the outcome was not documented on subsequent follow-up (49).

## 3 Discussion

This review highlights ultrasound-guided peripheral nerve blocks targeting joint-specific nociceptive pathways as a minimally invasive option for pain management in OA.

A key distinction, often blurred in the literature, is that between diagnostic/prognostic blockade — performed to confirm the pain source before a longer-lasting intervention such as ablation — and therapeutic blockade, which is the focus of the present review; the included studies predominantly assessed the analgesic and functional effect of the block itself rather than its predictive value for a subsequent procedure.

All four blocks share the rationale of selectively interrupting articular nociceptive transmission while preserving motor function, yet the quality of supporting evidence varies considerably across blocks. The genicular nerve block has the most robust evidence base, supported by placebo-controlled RCTs and meta-analyses. The SSN block is supported by one placebo-controlled RCT in degenerative shoulder disease and a meta-analysis of broader chronic shoulder pain populations. The PENG block has only one RCT comparing it with an active comparator; and the DPN block remains limited to retrospective case series with no controlled trials. Table 1 summarizes the comparative evidence across the four nerve blocks reviewed.

**Table 1:** Summary of evidence across ultrasound-guided peripheral nerve blocks for osteoarthritis.

Nerve block	Study	Design	*N*	Population	Intervention	Comparator	Key outcomes	Follow-up
Genicular	Shanahan et al. 2023 (17)	Placebo-controlled RCT	64 patients	Knee OA	Bupivacaine + betamethasone	Saline placebo	VAS pain improved at weeks 2,4, 8 but effect attenuated by week 12; WOMAC improved week 12	12 weeks
Genicular	Binici et al. 2026 (21)	Placebo-controlled RCT	81 patients	Knee OA	G1: Prilocaine; G2: Prilocaine + triamcinolone	Saline placebo	VAS improved in both active groups at 1 week and 1 month; WOMAC improved significantly active groups by 1 month; no difference G1 vs G2	1 month
Genicular	De la Fuente-Escudero et al. 2026 (20)	Placebo-controlled RCT	102 patients	Knee OA	G1: Bupivacaine; G2: Bupivacaine + triamcinolone	Saline placebo	NRS pain reduction sustained at 24 weeks; WOMAC function improved sustained at 24 weeks only in G1	24 weeks
Suprascapular	Shanahan et al. 2003 (34)	Placebo-controlled RCT	83 (108 shoulders)	Chronic shoulder pain (52% degenerative + 48% RA)	Bupivacaine + methylprednisolone	Saline placebo	SPADI pain and disability improved at weeks 1, 4, and 12	12 weeks
PENG	Kose et al. 2023 (43)	RCT	60 patients	Hip OA	Bupivacaine + dexamethasone	Intra-articular injection (same injectate)	PENG superior at day 1 in NRS; IA superior at week 4 and 8 in pains, HHS and WOMAC	8 weeks
Deep peroneal	Shi et al. 2019 (48)	Retrospective series	37 patients	Midfoot OA	Lidocaine + bupivacaine	—	Mean immediate pain improvement 92.1%	Immediate
Deep peroneal	Johnston et al. 2020 (49)	Retrospective series	25 feet	Midfoot OA (88%)	Lidocaine + bupivacaine	—	Pain relief in 88%	Immediate or subsequente visit

G1, Group 1; G2, Group 2; IA, intra-articular; NRS, numeric rating scale; VAS, visual analogue scale; WOMAC, Western Ontario and McMaster Universities Osteoarthritis Index; SPADI, shoulder pain and disability index; HHS, Harris Hip Score; OA, osteoarthritis; RA, rheumatoid arthritis.

Several cross-cutting limitations emerge from this review. Follow-up duration is limited across all blocks, with a maximum of 24 weeks in the genicular block (20), 12 weeks for the SSN (34) and 8 weeks for the PENG block (43), while DPN block lacks any structured follow-up beyond immediate post-procedural assessment (48, 49). Substantial heterogeneity exists across studies in guidance technique, comparator interventions, injectate regimens (including agent, concentration and volume), outcome measures, and patient populations, limiting direct comparison and precluding definitive conclusions on optimal protocols.

To our knowledge, serial or repeated blocks in OA have only been described anecdotally (50, 51), and no controlled study has defined optimal re-injection intervals or long-term outcomes beyond a single administration for any of the four blocks reviewed.

The role of corticosteroid as an adjuvant to local anesthetic remains unresolved. For the genicular block, three RCTs found no sustained advantage of adding corticosteroid (20, 21, 24). In the de la Fuente-Escudero et al. trial, functional improvement at 24 weeks tended to be more sustained in the local anesthetic-alone group (20); although no direct comparison between the two active arms was reported, this reinforces the absence of any added benefit from corticosteroid reported in other RCTs. Given the potential adverse effects of repeated corticosteroid exposure, the available evidence does not support its routine use in GNB for knee OA. For the SSN block, indirect evidence suggests a possible advantage of combined regimens, though no head-to-head trial exists (36). For the PENG block, both arms of the only available RCT included dexamethasone, precluding any comparison (43). No data exist for the DPN block. The type of corticosteroid has likewise not been examined. Where specified, studies of the genicular and suprascapular blocks used particulate agents (triamcinolone and methylprednisolone), whereas the only OA-specific trial of the PENG block used dexamethasone in both arms, precluding comparison (21, 24, 34, 36, 43). Perineural dexamethasone has been shown in the perioperative setting to prolong single-injection blocks (41), but whether this translates into sustained benefit in chronic OA pain is unknown, and no study has directly compared particulate with non-particulate agents in any of the blocks reviewed.

The quality of adverse event assessment and reporting is generally poor across the literature for all four blocks. The most comprehensive safety data come from a systematic review of 111 SSN block studies, highlighting substantial deficiencies in harms assessment methodology (38)**.**

The evidence for GNB in knee OA, while growing, has important limitations. A GRADE-based meta-analysis rated the overall certainty of evidence as very low to low, citing small trial sizes, risk of bias, and inconsistent effect estimates (23). The duration of analgesic effect varies across studies, with RCTs reporting significant pain relief up to 24 weeks (20–22).

Direct evidence for the SSN block in glenohumeral OA is scarce. The only placebo-controlled RCT enrolled patients with chronic shoulder pain from OA (both degenerative and rheumatoid), rather than a population selected specifically for primary glenohumeral OA (34). All remaining evidence—including Chang et al. meta-analysis (35) and the Scattergood et al. systematic review (36)—derives from heterogeneous chronic shoulder pain populations, and the extrapolation to glenohumeral OA rests primarily on the anatomical rationale. The analgesic effect appears sustained up to 12 weeks (34–36). Regarding technique, the posterior fossa approach is preferred over the supraclavicular approach due to a substantially lower risk of phrenic nerve blockade (32), and ultrasound guidance appeared to yield greater effect sizes than landmark-based approaches across all timepoints in the Chang et al. meta-analysis (35), further supporting imaging-guided techniques.

Evidence for the PENG block in hip OA remains at an early stage, with no meta-analysis or systematic review currently available for this indication. The only RCT in OA demonstrated superior early analgesia with the PENG block (day 1), but the intra-articular corticosteroid group achieved better pain and functional outcomes at weeks 4 and 8 (43). The clinical significance of this temporal pattern requires confirmation in larger trials with longer follow-up. The motor-sparing profile — the principal proposed advantage over femoral nerve and fascia iliaca blocks — appears volume-dependent, with motor preservation reported at lower volumes but quadriceps weakness documented at higher volumes (≥20 mL), suggesting that volume optimization warrants further study (42).

The evidence for DPN blockade in OA is the most limited among the four blocks reviewed, consisting entirely of small retrospective series without controlled comparisons, largely in midfoot rather than talocrural OA, leaving its role in ankle OA undefined. Its current role remains primarily diagnostic — confirming that dorsal midfoot pain is at least partially DPN-mediated — with its therapeutic potential as a standalone intervention still unproven, although the consistently high rates of immediate pain relief support a strong anatomical rationale for targeting this nerve (48, 49). Midfoot OA has limited surgical options and scarce evidence for any non-surgical intervention (52), which may increase the clinical relevance of a nerve block approach in this population but also underscores the urgent need for prospective controlled trials.

Where active comparators were used, no consistent pattern emerged across targets: in the shoulder, SSN blockade was superior to physical therapy but no different from intra-articular corticosteroid injection (35); in the knee, small differences favored genicular blockade over both comparators (22, 23); and in the hip, the intra-articular comparator performed better from week 4 onwards (43). No comparison demonstrated a clear advantage of nerve blockade over established interventions, and the underlying evidence is limited and of low quality throughout.

Beyond reversible blockade, longer-lasting denervation strategies targeting the same articular nerves are being explored for OA pain. For the knee, a meta-analysis of randomized trials comparing ablation techniques with sham reported significant pain reduction at 4, 12 and 24 weeks, with a diminishing effect over time (53). Thermal and chemical ablation have also been applied to the other joints: continuous radiofrequency lesioning of the SSN at the spinoglenoid notch, using a motor-sparing technique, was associated with significant pain reduction in patients with glenohumeral OA (54), and ethanol neurolysis at the PENG target reduced pain up to 6 months in a sham-controlled RCT (55); more broadly, a systematic review of nerve-targeted interventions for refractory hip pain, including OA — predominantly radiofrequency ablation targeting the FN and obturator nerves — reported >50% pain relief in 60%–80% of patients at 3–6 months, although almost entirely from small non-comparative series (56). More recently, cryoneurolysis has been described as a motor-sparing approach in the shoulder, targeting individual articular branches, in a small case series of patients with rotator cuff disease rather than OA (57). In ankle OA, an open-label trial of cryoneurolysis targeting the DPN among other nerves improved pain and function up to 24 weeks (58). In several of these studies, procedures are typically preceded by a diagnostic block to confirm the pain source, positioning peripheral nerve blockade as a selection step within a broader interventional pathway. Ablative approaches fall outside the scope of this review but represent a relevant direction for future research.

This review offers a structured, multi-joint overview of ultrasound-guided peripheral nerve blocks in OA, applying a consistent framework across four articular targets and enabling cross-comparison of the maturity of evidence for each. The inclusion of recent RCTs published in 2025–2026 not yet captured by existing meta-analyses provides an updated perspective on the evidence base.

A critical appraisal approach — distinguishing direct OA-specific evidence from indirect data extrapolated from broader chronic pain populations — was maintained throughout.

Several limitations should be acknowledged. This is a narrative review, not a systematic review, and selection bias in study inclusion cannot be excluded. The underlying evidence is predominantly of low to very low quality, characterized by small sample sizes, single-center designs, and heterogeneous procedural techniques, injectate regimens, comparator groups, and outcome measures, which complicates direct comparison across studies and blocks. RCTs remain scarce — particularly for SSN, PENG, and DPN blocks — and long-term data on durability of benefit, need for repeat injections, and cumulative safety are lacking, with follow-up rarely exceeding 24 weeks. For the SSN block, most evidence derives from mixed chronic pain populations rather than OA-specific cohorts, requiring caution in extrapolation. For the DPN block, the absence of any controlled trial precludes conclusions about therapeutic efficacy. The available data do not allow conclusions about whether the magnitude of benefit differs between patients with moderate and severe baseline pain. Finally, the search was limited to English-language publications, potentially introducing linguistic bias.

In conclusion, ultrasound-guided peripheral nerve blocks targeting joint-specific nociceptive pathways represent a minimally invasive option that may provide analgesic benefit as part of a multimodal approach in selected patients with refractory OA pain, but should currently be regarded as investigational rather than an established treatment. The genicular nerve block has the most developed evidence base, with multiple placebo-controlled RCTs demonstrating significant pain relief, for up to 24 weeks in the longest trial. The suprascapular and PENG blocks are supported by limited but encouraging data, while the DPN block remains at a preliminary, diagnostic stage. Across all four blocks, the available evidence consistently demonstrates a favorable safety profile with preservation of motor function. However, the positioning of peripheral nerve blocks within the OA treatment algorithm — how it compares with established options such as intra-articular injection, and whether the blocks may serve as a step preceding ablative procedures or surgery — remains to be defined. Well-designed sham-controlled RCTs with standardized protocols, validated outcome measures, and extended follow-up are needed to establish the efficacy, optimal technique and durability of benefit of these interventions across different joints and stages of OA.
